# Social assistance programme impacts on women's and children's diets and nutritional status

**DOI:** 10.1111/mcn.13378

**Published:** 2022-06-20

**Authors:** Deanna K. Olney, Aulo Gelli, Neha Kumar, Harold Alderman, Ara Go, Ahmed Raza

**Affiliations:** ^1^ Poverty, Health and Nutrition Division International Food Policy Research Institute (IFPRI) Washington DC United States; ^2^ Food and Agriculture Organization of the United Nations (FAO) Rome Italy

**Keywords:** children, dietary diversity, micronutrient intake, nutritional status, social assistance, social protection, women

## Abstract

Investments in social assistance programmes (SAPs) have accelerated alongside interest in using SAPs to improve health and nutrition outcomes. However, evidence of how design features within and across programme types influence the effectiveness of SAPs for improving diet and nutrition outcomes among women and children is limited. To address this, we reviewed evaluations of cash, in‐kind and voucher programmes conducted between 2010 and 2020 among women and children, and examined associations between design features (targeting, including household and individual transfers, fortified foods and behaviour change communication) and positive impacts on diet (diet diversity, micronutrient intake) and nutrition (anthropometric indicators, haemoglobin, anaemia) outcomes. Our review has several key findings. First, SAPs improve dietary diversity and intake of micronutrient‐rich foods among women and children, as well as improve several nutrition outcomes. Second, SAPs were more likely to impact diet and nutrition outcomes among women compared with children (23/45 [51%] vs. 52/144 [36%] of outcomes measured). Third, in‐kind (all but one of which included fortified foods) compared with cash transfer programmes were more likely to significantly increase women's body mass index and children's weight‐for‐height/length *Z*‐score, and both women's and children's haemoglobin and anaemia. However, there is limited evidence on the effectiveness of SAPs for improving micronutrient status and preventing increased prevalence of overweight and obesity for all populations and for improving diet and nutrition outcomes among men, adolescents and the elderly. Further research in these areas is urgently needed to optimize impact of SAPs on diet and nutrition outcomes as countries increase investments in SAPs.

## Key Points/Highlights

- Social assistance programmes (SAPs) can be used to improve diets of women and children.
- Effectiveness of SAPs may differ by programme type and outcome assessed. For example, in‐kind transfers (with fortified foods) compared with cash transfer programmes were more likely to significantly increase children's weight‐for‐length/height *Z*‐score (WLZ/WHZ), and women's and children's haemoglobin concentration (Hb) and decrease anaemia prevalence.
- If programmes aim to reduce stunting and anaemia or increase WLZ/WHZ, mid‐upper arm circumference or Hb, they should consider targeting women and/or young children, including both household and individual transfers, fortified foods/supplement and/or behaviour change communication.
- Evidence of the effectiveness of SAPs for improving micronutrient status and preventing an increase in overweight and obesity is scarce as is evidence for the effectiveness of SAPs for improving diet and nutrition outcomes among men, adolescents and the elderly in low‐ and middle‐income countries.
- To better understand which programme design features are most important for achieving impacts, randomized evaluation designs and consistency in programme and evaluation design across several programmes in different contexts are needed.

## 1 INTRODUCTION

Investments in social assistance programmes (SAPs) have accelerated rapidly in recent years. Between 2000 and 2016, the share of gross domestic product spent on these types of programmes tripled. For low‐ and middle‐income countries, about 1.5% of gross domestic product is spent on SAPs (World Bank, 2018); moreover, this increased due to the COVID‐19 pandemic. SAPs are primarily designed to address the income constraints of the poor by providing transfers of cash, food, vouchers or assets to help them meet their basic needs, support their livelihoods and increase their resilience. The primary goal of most SAPs is to reduce poverty. However, they have the potential to do more to achieve other important social goals for those most in need.

In the past decade, there has been growing interest in using SAPs to improve health and nutrition outcomes. To achieve these goals, many programmes have included one or more specific design features, such as the following: (1) targeting the most nutritionally vulnerable members within poor households such as pregnant and lactating women or young children; or (2) including a nutrition‐specific intervention for vulnerable groups within households, in addition to the household‐level support; or (3) integrating nutrition‐specific interventions, more generally, such as the provision of fortified foods or inclusion of health and nutrition behaviour change communication (BCC) activities (Olney et al., 2019; Ruel & Alderman, 2013). Moreover, some SAPs include conditionalities to encourage use of services such as preventive health services. SAPs that are designed in this way can be described as being nutrition‐sensitive, meaning that they are designed with nutrition in mind, have specific nutrition goals and actions in place to achieve those goals, and simultaneously address the basic and underlying causes of malnutrition such as poverty, inadequate access to food and health care, inadequate access to safe and hygienic environments, and suboptimal care practices (Ruel & Alderman, 2013).

Although there is an increase in the use of SAPs to address both food security and nutrition outcomes, the evidence of their impact on nutrition outcomes is limited (Alderman, 2016). Various reviews have assessed the average impact of cash transfers using meta‐analyses, most recently in Manley et al. (in press). The conclusions from these have indicated the average impact of such programmes on a limited set of outcomes but, unfortunately, the data base is too limited to quantify any heterogenous impacts across programme types and implementation approaches. In particular, due to the wide variety of programme designs, it has been challenging to determine how these types of programmes work to achieve nutrition outcomes and what components or combination of components need to be in place to achieve optimal impact for a given cost. Understanding this is essential for leveraging investments in SAPs to meet poverty reduction, improved food security and nutrition goals simultaneously as well as to enable cost‐effective scaleup of successful programmes.

An understanding of impacts and costs across different types of SAPs and design features associated with positive impacts across a range of diet and nutrition outcomes could support policymakers in determining what types of SAPs or combination of SAPs would be best suited for their context. In this study, we present evidence from a review of the impact of SAPs on diet and nutrition outcomes among women and children. We assess the evidence of impact within and across three types of SAPs—cash transfers, in‐kind transfers and voucher programmes—for a range of diet and nutrition outcomes among women and children. In addition, we qualitatively assessed how different programme design features may be contributing to the success of SAPs to improve nutrition outcomes. Lastly, we identify key evidence gaps.

## 2 METHODS

### 2.1 Search strategy

An electronic search was carried out using ISI Web of Science in June 2020 to search for articles published between 2010 and 2020, with no language restrictions. We searched for general social protection programmes and by each of the main programme types including cash, in‐kind and vouchers. In each search, we included AND nutrition AND several specific diet and nutrition outcomes separated by OR to refine our search to evaluations of programmes that included at least one nutrition outcome assessed at the individual level. The full set of search terms used are listed in Table S1.

### 2.2 Inclusion/exclusion criteria

To be included in this review, studies had to be published in peer‐reviewed journals between 2010 and 2020, include at least one diet or nutrition outcome at the individual level, and have at least two time points and a valid counterfactual to establish causality between interventions and outcomes. For diet‐related outcomes, we considered dietary diversity, micronutrient intake, as well as infant and young child feeding practices. Nutrition outcomes included anthropometric measures, anaemia, and micronutrient status. Studies were excluded if they were review studies, observational studies with only one time point or were conducted in high‐income countries.

### 2.3 Screening

An initial screening of titles and abstracts was performed to refine the list of studies that would be fully reviewed. This yielded 166 papers out of 558 publications initially identified. These papers were reviewed and included or excluded based on the criteria listed above. In addition, the co‐authors and our partners at XXX reviewed the list and included missing publications based on their respective expertise. After the full review process, 36 papers were ultimately included and together represent 32 unique programme evaluations and 48 unique study arms (Table S2).

### 2.4 Analysis

To summarize the evidence, we conducted two sets of qualitative analyses. For both sets of analyses we used individual treatment arms as the unit of analysis. Each treatment arm represents a different intervention, implementation or targeting approach and, thus, is unique in its potential to improve diet and nutrition outcomes.

We first assessed the proportion of treatment arms with positive, neutral or negative impacts on diet and nutrition outcomes for women and children within each of the different programme types and then across all programme types. The primary diet‐related outcomes for which we summarized findings include dietary diversity and micronutrient intake. Dietary diversity included outcomes related to dietary diversity score or minimum dietary diversity for women or children. Micronutrient intake included outcomes related to actual micronutrient intake or intake of micronutrient‐rich food such as reported intake of iron‐rich and/or vitamin A‐rich foods. The primary nutrition status outcomes for which results were summarized include height‐for‐age *Z*‐score (HAZ), stunting (HAZ < −2), weight‐for‐length/height *Z*‐score (WLZ/WHZ), wasting (WHZ < −2), body mass index (BMI), underweight among adults (BMI < 18.5 kg/m^2^), overweight or obesity (BMI > 25 kg/m^2^ or BMI > 30 kg/m^2^, respectively), mid‐upper arm circumference (MUAC), haemoglobin concentration (Hb), anaemia (Hb < 11 g/dl) and micronutrient status. For this part of the analyses, we grouped the proportion of positive impacts into four categories (<10% was considered unlikely to have positive impact; 10–25% was considered to have limited potential for impact; 26–49% considered to have potential for impact; ≥50% was considered to have positive impact). For this analysis, if outcomes had less than three study arms, we categorized these as not having enough information.

The second part of the analysis was designed to better understand the programme design features within SAPs associated with positive dietary and nutritional status impacts among women and children. To do this, we assessed programme impact according to whether different design features were included or not. For these analyses design features were selected based on their potential for influencing the diet or nutrition outcomes assessed. For impacts on women's dietary diversity and BMI, we included the following design features: whether the treatment arms were targeted to women, whether they included BCC and whether they provided both individual and household transfers. For impacts on children's dietary diversity and micronutrient intake, we assessed impact by whether treatment arms were targeted to women and/or children, whether they included BCC and provided both individual and household transfers. For impacts on LAZ/HAZ, stunting, WLZ/WHZ, wasting, MUAC, Hb and anaemia among children, we assessed impact by the aforementioned features as well as by whether the treatment arms included a fortified food. Within each of these categories, we assessed the proportion of study arms that had positive or neutral/negative impacts. Neutral and negative impacts were combined as very few negative impacts were found. We conducted this second set of analyses only for outcomes that were assessed in at least eight treatment arms within a population group (e.g., diet diversity among women or anaemia among children). Further we only considered differences between whether a design feature was included or not if there were at least two treatment arms in each condition (i.e., had and did not have the design feature). Group differences were considered to be potentially meaningful if the difference in proportion of positive impacts between treatment arms that had the design feature versus those that did not was at least 10 pp.

## 3 RESULTS

Our review resulted in the inclusion of 32 SAP evaluations, with 48 unique treatment arms (Table S2). Evaluations of cash transfer programmes were the most common (18 evaluations and 23 treatment arms) followed by in‐kind (10 evaluations and 21 treatment arms) and voucher programmes (four evaluations and four treatment arms). Most of the studies were conducted in either Africa or Latin America and the Caribbean (Table S3). The details related to the programme design features included, and diet and nutrition outcomes assessed among women and children within each programme type (cash, in‐kind and vouchers) can be found in Tables 4 through 9.

Overall, among women, anthropometric assessments were the most commonly assessed diet or nutrition outcomes, with BMI being the most commonly assessed outcome followed by MUAC. Diet diversity was assessed in nine treatment arms and micronutrient intake in three treatment arms (one each from each type of SAP). Lastly, Hb and anaemia were assessed in seven treatment arms with the majority of the treatment arms being part of cash or in‐kind transfer programmes (Table 1).

**Table 1:** Proportion of study arms with positive impact on outcomes by programme type among women^†^

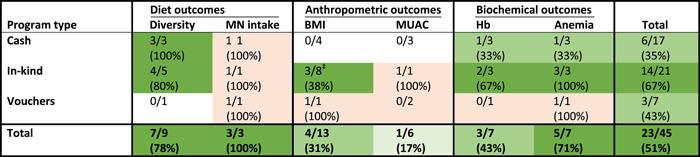

Abbreviations: BMI, body mass index; Hb, haemoglobin; MN, micronutrient; MUAC, mid‐upper arm circumference.

^†^No colour, <10% considered unlikely to have a positive impact. Light orange, not enough information. Lightest green, 10–25% considered to have limited potential for positive impact. Medium green, 26–49% considered to have potential for positive impact under certain conditions. Darkest green, ≥50% considered likely to have a positive impact.

^‡^In the one study with an increase in BMI (three treatment arms), the increase in BMI (considered positive impact here) are likely unintentional negative effects given that this programme was implemented in Guatemala where there is a high prevalence of overweight and obesity.

The impact on BMI was examined by the greatest number of treatment arms (*n* = 13) and of these four treatment arms across three studies found significantly higher BMI compared with control. However, in two of the study contexts (four treatment arms providing in‐kind transfers) the prevalence of overweight and obesity is high (Mexico and Guatemala) and, thus, an increase in BMI is likely an unintentional negative programme impact. However, increased overweight/obesity as measured by BMI cutoffs was not investigated in any of the studies identified. One study in Mexico did assess the impact of the programme on waist circumference and found a negative effect of the programme that include food transfers alone compared with control (but not in the group that included food + BCC compared with control) on increasing the proportion of women with waist circumference > 88 cm, which is indicative of abdominal obesity (Avitabile, 2012). The impact of SAPs on MUAC was examined by six treatment arms. One study with two treatment arms (cash + participatory learning approach [PLA] and in‐kind + PLA) reported positive impacts on MUAC in pregnancy (Harris‐Fry et al., 2018). However, this positive impact in the treatment arm that included in‐kind transfers and PLA, was not maintained postpartum (Saville et al., 2018). Neither of the evaluations of voucher programmes that assessed impact on MUAC among women found a positive impact (Table 1).

Micronutrient intake was improved in all treatment arms in which it was assessed and diet diversity was increased in seven out of the nine treatment arms in which it was assessed. These results indicate significant, consistent impacts of SAPs on diet‐related outcomes.

More than half of the seven treatment arms that aimed to decrease anaemia prevalence among women were successful in doing so. However, only three of the seven treatment arms found a similar positive impact on increasing Hb. One study, with three treatment arms that assessed the impact of the Preventing Malnutrition in children under 2 years of Age (PM2A) programme (provision of fortified food, BCC and encouragement to attend preventive health visits), found strong consistent positive impacts on reducing anaemia and increasing Hb among women up to 3 months postpartum when the risk of anaemia is very high (Leroy et al., 2016).

Among children, we summarized programme impacts across the same three SAPs as for women. As was the case for women's outcomes, evaluations of cash and in‐kind transfer programmes were the most common. Among children, anthropometric outcomes were the most frequently assessed with the most common outcomes assessed being LAZ/HAZ and stunting followed by LHZ/WHZ and wasting (Table 2). Several studies also assessed programme impact on children's diets and on MUAC, Hb and anaemia. Only one study assessed programme impact on children's micronutrient status specifically and, thus, this is not elaborated upon in this review.

**Table 2:** Proportion of study arms with positive impact on outcomes by programme type among children^†^

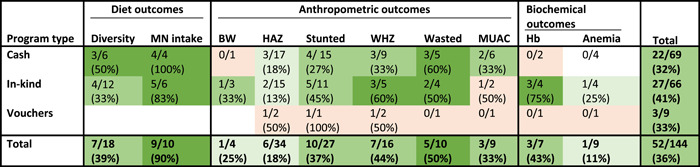

Abbreviations: BW, birth weight; HAZ, height‐for‐age Z‐score; Hb, haemoglobin; MN, micronutrient; MUAC, mid‐upper arm circumference; WHZ, weight‐for‐height *Z*‐score.

^†^No colour, <10% considered unlikely to have positive impact. Light orange, not enough information. Lightest green, 10–25% considered to have limited potential for positive impact. Medium green, 26–49% considered to have potential for positive impact. Darkest green, ≥50% considered likely to have a positive impact.

SAPs improved diet and nutritional status outcomes among children in about one‐third of the outcomes assessed across the different programme types (Table 2). Within programme types, in‐kind programmes found positive impacts on 27/66 (41%) of the outcomes assessed, while both cash and voucher programmes found positive impacts for about one‐third of outcomes assessed (22/69 [32%] and 3/9 [33%], respectively). However, for vouchers, the positive impacts came from only one evaluation of a voucher programme implemented in Pakistan where positive impacts on HAZ, stunting and WHZ were found (Fenn et al., 2017; Table S8).

Similar to what we observe among women, impacts on children's diet outcomes were largely positive. Specifically, impacts on micronutrient intake or intake of micronutrient‐rich foods were the most likely to be positive followed by dietary diversity (improved in 9/10 [90%] and 7/18 [39%] of treatment arms, respectively; Table 2). None of the evaluations of voucher programmes included in this review assessed impact on children's dietary outcomes.

Overall, a large number of studies examined impacts on anthropometric outcomes among children. The two most commonly studied outcomes among the studies included in this review were programme impacts on LAZ/HAZ and prevalence of stunting, which has been a key focus for achieving improved nutrition among children for many low‐ and middle‐income countries in the past decade. There were 34 treatment arms that examined impacts on LAZ/HAZ, but only 6/34 (18%) resulted in a positive impact on this indicator. A larger share of the treatment arms 10/27 (37%) found significant positive impacts on reducing the prevalence of stunting (Table 2). Half of the positive impacts on stunting reduction were found in the arms in evaluations of PM2A programmes implemented in Burundi and Guatemala where in‐kind transfers that included fortified foods and BCC were provided monthly and use of preventive health visits encouraged, throughout the 1000‐day period (from pregnancy through 2 years of age; Leroy et al., 2018; Olney et al., 2018). The success rate in improving WHZ and wasting prevalence was higher compared with that for HAZ and stunting. Out of the 16 treatment arms that assessed impact on WLZ/WHZ, 7/16 (44%) reported positive impacts. About half of the treatment arms in the in‐kind (3/5 [60%]) and vouchers group (1/2 [50%]) found positive impacts on WLZ/WHZ, whereas only one‐third of those of the treatment arms in the cash programmes (3/9 [33%]) found positive impacts. Five out of the 10 treatment arms in which impacts on wasting were assessed reported reductions with all of the positive impacts found in evaluations of cash and in‐kind programmes. Nine treatment arms reported on MUAC among children and three of these reported positive impacts. Four treatment arms, across three studies, assessed programme impact on birth weight and prevalence of low birth weight, three of these treatment arms (from two studies) provided in‐kind transfers. Only one of these treatment arms improved birth weight (Table 2) and none had a positive impact on the prevalence of low birth weight. The one programme in which a positive impact was found was an in‐kind transfer programme, which provided fortified foods to pregnant women and support through a PLA implemented in Nepal (Saville et al., 2018).

Impacts on children's Hb and anaemia prevalence were assessed in each of the programme types included in this review, although the total number of studies is modest. Across all programme types three of the seven and one of the nine treatment arms that assessed impact on Hb and anaemia prevalence, respectively, found positive impacts. Positive impacts were limited to treatment arms within the in‐kind programme group and were limited to positive programme impacts from the PM2A programme in Burundi. Among the in‐kind treatment arms three out of four improved Hb and one out of four reduced anaemia prevalence. None of the treatment arms in the cash or vouchers programmes resulted in positive impacts on either children's Hb or anaemia prevalence (Table 2).

### 3.1 Exploring heterogeneity by programme design feature

Among women, SAPs were most effective at increasing dietary diversity when they included BCC and/or household and individual transfers (Table 3). Although 7/8 (88%) of the treatment arms that targeted women had positive impacts on dietary diversity, only one treatment arm did not target women in an in‐kind transfer programme, which was targeted to food insecure adults who recently started receiving antiretroviral therapy (Fahey et al., 2019) and thus we cannot draw conclusions on targeting for this outcome. Nearly all of the treatment arms within the programme evaluations included in this review included a BCC component. However, of those that did not include BCC, a lower proportion of treatment arms find a positive impact on dietary diversity as compared to those that did include BCC (2/3 [67%] vs. 5/6 [83%]; Table 3). Among the five treatment arms that did not include both individual and household transfers only three had positive impacts on women's dietary diversity—all of which provided cash transfers. Specifically, these were the programmes implemented in Burkina Faso (Houngbe et al., 2019) and Somalia (Grijalva‐Eternod et al., 2018), which included nutrition‐sensitive features that may have contributed to the programmes’ impacts on women's dietary diversity.

**Table 3:** Proportion of study arms with positive impact on women's dietary diversity by programme feature and programme type^†^

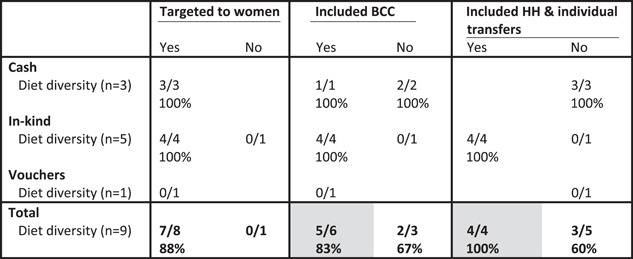

Abbreviations: BCC, behaviour change communication; HH, household.

^†^Grey shading, at least two treatment arms in each condition (yes/no) and difference between conditions ≥10 pp.

Among women, treatment arms that were targeted to women, included BCC and included both household and individual transfers were more likely to increase BMI than those that did not (Table 4). For example, treatment arms that were targeted to women (*n* = 8) were also more likely than those that were not (*n* = 5), to increase women's BMI. Only two treatment arms did not include BCC and neither of them led to an increase in BMI (Table 4). About half of the treatment arms provided both individual and household transfers. Of those that did, three (all in‐kind transfer treatment arms) of the six (50%) led to significant increases in women's BMI, whereas only one (a voucher programme in Pakistan [Fenn et al., 2017]) of the seven (14%) did not led to an increase in women's BMI (Table 4). All of the treatment arms that increased women's BMI were from two in‐kind transfer programmes implemented in Guatemala (Leroy et al., 2019), where it is likely that the increase in BMI was an unintentional negative programme impact. The other programme that increased women's BMI was a voucher programme that was not directly targeted to women, implemented in Pakistan (Fenn et al., 2017).

**Table 4:** Proportion of study arms with positive impact on women's BMI by programme feature and programme type^a^

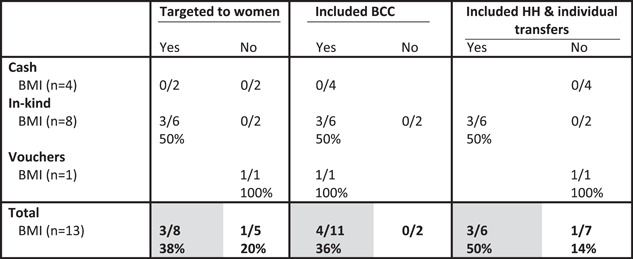

Abbreviations: BCC, behaviour change communication; BMI, body mass index; HH, household.

^a^ Grey shading, at least two treatment arms in each condition (yes/no) and difference between conditions ≥10 pp

Similar results were found in regard to increasing Hb and decreasing anaemia prevalence among women. Among the included studies, treatment arms that targeted women and included both household and individual transfers were associated with a higher proportion of positive impacts as compared to treatment arms that did not include those design features. The inclusion of fortified foods (or other products) was associated with a slightly higher proportion of positive impacts on anaemia but not for Hb (Table 5).

**Table 5:** Proportion of study arms with positive impact on women's Hb and anaemia by programme design features and programme type^†^

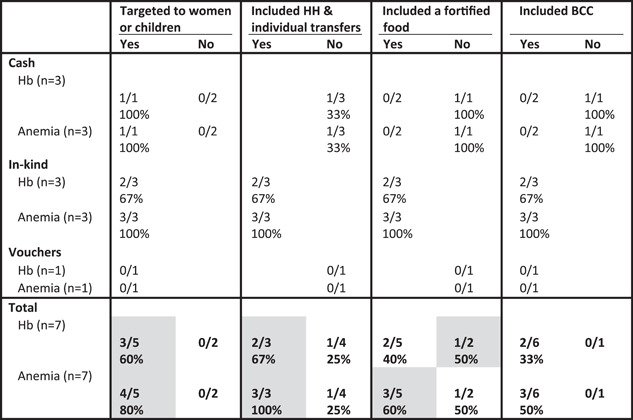

Abbreviations: BCC, behaviour change communication; BMI, body mass index; HH, household.

^†^Grey shading, at least two treatment arms in each condition (yes/no) and difference between conditions ≥10 pp.

Among children, the impact of SAPs on diet diversity was more commonly found in treatment arms targeted to women or children than those that were not (6/14 [43%] vs. 1/4 [25%]). However, treatment arms that did not include BCC and that did not include both household and individual transfers were more likely to increase diet diversity than those that did include those programme components (Table 6). In‐kind transfer programmes were heterogeneous in the relative importance of the different design features. For these programmes targeting women and children (3/8 [38%] vs. 1/4 [25%]) and including both household and individual transfers (2/5 [40%] vs. 2/7 [29%]) were associated with a greater proportion of positive impacts on dietary diversity whereas, the inclusion of BCC was associated with a smaller proportion of positive impacts (2/7 [29%] vs. 2/5 [40%]; Table 6). Nine out of 10 treatment arms increased micronutrient intake or intake of micronutrient‐rich foods, so it is difficult to draw conclusion regarding relative importance of different design features for this outcome.

**Table 6:** Proportion of study arms with positive impact on children's dietary diversity and micronutrient intake by design feature and programme type^†^

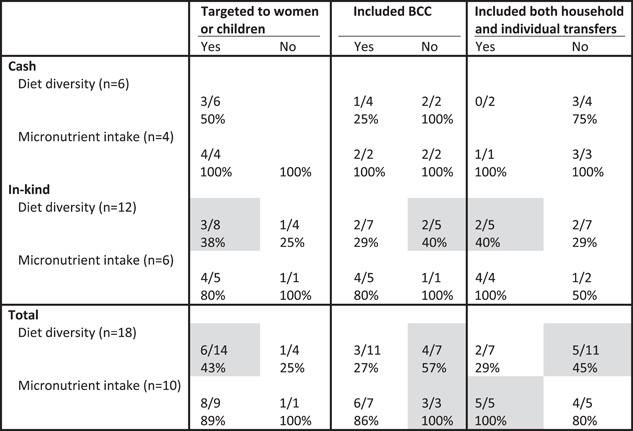

Abbreviation: BCC, behaviour change communication.

^†^Grey shading, at least two treatment arms in each condition (yes/no) and difference between conditions ≥10 pp.

Impacts on HAZ and stunting were most commonly assessed in the evaluation of cash and in‐kind transfer programmes. Across the different programme types, a clear pattern of associations emerged for a positive impact on reducing stunting. Specifically, those that targeted women or children (10/24 [42%] vs. 0/3), included both individual and household transfers (5/11 [45%] vs. 5/16 [31%]), included fortified foods (7/15 [47%] vs. 3/12 [25%]) and a BCC component (9/18 [50%] vs. 1/9 [11%]) were more likely to significantly reduce stunting compared with those that did not include those components. For HAZ, the most important design feature appeared to be targeting women or children. The inclusion of both a household and individual ration, fortified foods nor BCC appeared to be associated with a higher proportion of positive impacts on LAZ/HAZ compared with those that did not have these design features (Table 7).

**Table 7:** Proportion of study arms with positive impact on children's HAZ and stunting (HAZ < ‐2) by programme design feature and programme type^†^

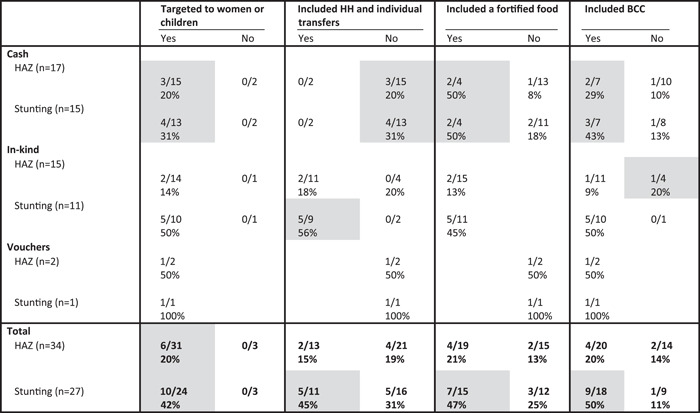

Abbreviations: BCC, behaviour change communication; HH, household; HAZ, height‐for‐age *Z*‐score

^†^Grey shading, at least two treatment arms in each condition (yes/no) and difference between conditions ≥10 pp.

Only about half as many study arms assessed impacts on increasing WHZ or reducing the prevalence of wasting as compared to those that assessed impact on linear growth. Of those that did some of the programmes were implemented as preventative programmes (implementation in areas at high risk of malnutrition), whereas others were implemented as curative approaches (targeting children once they become malnourished). As was common for study arms that assessed impact on children's diet and nutrition outcomes, most targeted women or children (Table 8). Including fortified foods and/or a BCC component was associated with positive programme impacts on WHZ, but the opposite was found for the prevalence of wasting where programmes that did not include fortified foods and/or BCC were more likely to have a positive impact on reducing wasting than those that included those design features. Given the opposite findings for these two closely related indicators, it is difficult to draw conclusions on the relative importance of these design features for increasing WHZ and reducing wasting (Table 8).

**Table 8:** Proportion of study arms with positive impact on children's WHZ and wasting (WHZ < −2) by programme design feature and programme type^a^

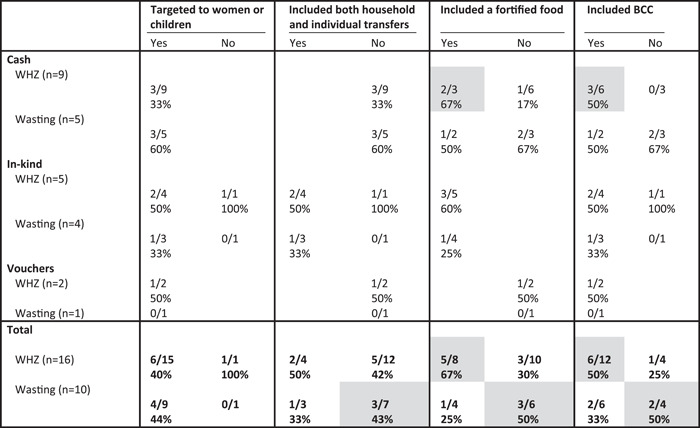

Abbreviations: BCC, behaviour change communication; WHZ, weight‐for‐height *Z*‐score.

^a^ Grey shading, at least two treatment arms in each condition (yes/no) and difference between conditions ≥ 10 pp.

Nine treatment arms assessed programme impact on MUAC and of the design features included in this analysis, inclusion of fortified foods and BCC were associated with a higher proportion of positive impacts compared to treatment arms that did not have these design features (Table 9). For this outcome, all treatment arms were targeted to women and only one included both a household and individual transfer and thus, we cannot draw conclusions for these two design features for this outcome.

**Table 9:** Proportion of study arms with positive impact on children's MUAC by programme design features and programme type^†^

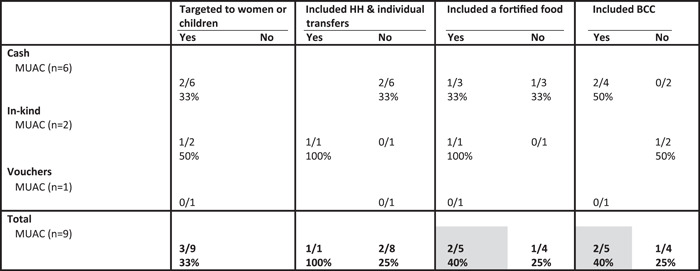

Abbreviations: BCC, behaviour change communication; HH, household; MUAC, mid‐upper arm circumference.

^†^Grey shading, at least two treatment arms in each condition (yes/no) and difference between conditions ≥10 pp.

Across the different types of SAPs included in this review, seven included assessments of impact on Hb and nine on anaemia and these were spread across the different programme types. Of the programme types, only treatment arms in the in‐kind programmes were effective for increasing Hb or reducing anaemia (Table 2). Within the in‐kind category, three of the four study arms led to a significant increase in Hb, but only one of four led to a significant decrease in the prevalence of anaemia (Table 10). The positive impacts were limited to three treatment arms in the PM2A programme in Burundi that was targeted to women and children, included both household and individual transfers of fortified foods and a BCC component.

**Table 10:** Proportion of study arms with positive impact on children's Hb and anaemia by programme design features and programme type^†^

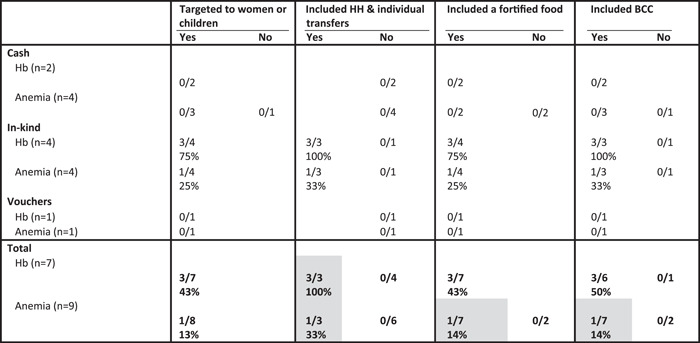

Abbreviations: BCC, behaviour change communication; Hb, haemoglobin; HH, household.

^†^Grey shading, at least two treatment arms in each condition (yes/no) and difference between conditions ≥10 pp.

## 4 DISCUSSION

This review of the impact of SAPs on women's and children's, and diet and nutrition outcomes revealed that, in general, SAPs are very effective for improving women's and children's dietary outcomes and can contribute to improving several nutrition‐related outcomes such as BMI, Hb, and anaemia among women, as well as stunting, WLZ/WHZ, wasting, MUAC and Hb among children. However, effectiveness for improving different types of outcomes, especially the nutrition‐related outcomes, varies substantially by programme type and may also vary by the inclusion or use of different programme design features such as targeting women or children, including both household and individual transfers, fortified foods, and BCC. Thus, when using SAPs to improve nutritional status outcomes, programme type along with the design features to be included, should be carefully selected based on the evidence to date.

In‐kind (all but one of which included fortified foods) and voucher programmes were more likely to lead to increases in BMI compared to cash transfer programmes. However, the increases in women's BMI may not always be a positive impact, but instead may be an unintentional negative impact in contexts with a high prevalence of overweight and obesity. This was found in the case of the evaluation of an in‐kind transfer programme in Guatemala. In the case of Guatemala, the programme was designed to decrease stunting prevalence among children in the first 1000 days primarily and was successful in doing so (Olney et al., 2018). However, it also led to higher BMI postpartum among women, which in the Guatemalan context may not be a positive impact (Leroy et al., 2019). When considering the programme design features associated with significant programme impacts, we also found that targeting women as well as including both household and individual transfers and a BCC component were associated with a higher proportion of study arms with an increase in BMI among women. Thus, where it is desirable to increase women's BMI such as in contexts where the prevalence of women's underweight is high and overweight and obesity is low, targeting women and including both household and individual transfers would likely be important for achieving that goal. On the other hand, where the prevalence of overweight and obesity is high, in‐kind transfer programmes should carefully consider what is included in the transfers (i.e., food and nutrient composition of the transfer) and include complementary BCC related to healthy eating and physical activity practices, or consider using a cash or voucher transfer with complementary BCC, so as to improve the targeted nutrition outcomes without leading to unintentional negative impacts on others, such as increasing women's BMI.

The vast majority of study arms included in this review either directly or indirectly targeted women or children. This may be a feature of study design and does not necessarily pertain to the general body of SAPs. The importance of this targeting approach was clear in our analysis where we found that when women or children were not targeted, there was little evidence of positive impact across the diet and nutrition‐related outcomes assessed. In the case of women's outcomes only one study arm in the context of a voucher programme that did not target women led to an increase in BMI. For children, we found a similar pattern, whereby only one study arm, an in‐kind transfer programme, that assessed programme impact on children's WHZ led to a positive impact. Programmes designed to improve women's and children's diets and nutrition‐related outcomes should continue to target women and/or children with the transfers. Clearer evidence on the role of direct (e.g., handing transfer to women) or indirect (providing messaging about the intended use of the transfers) transfers and control over and use of them once in the household, is needed, however, to further clarify how targeting can influence programme effectiveness.

Control over the use of the transfers is likely a salient issue. For example, in the PM2A programme in Burundi, which was effective in increasing several diet and nutrition‐related outcomes among women and children, women received their transfers at a monthly distribution site. A process evaluation conducted with this programme revealed that although the targeted women generally collected the transfers themselves, in some cases they were assisted by other household members to help carry the large quantity of food home. Despite sometimes receiving assistance to carry the food home, about 90% of the women interviewed said that they dictated how the food provided by the programme was used (Olney et al., 2013).

The inclusion of BCC is often regarded as a key component for programmes aiming to improve nutrition outcomes. In our review, among women, study arms that included BCC were more likely to lead to increases in dietary diversity and BMI. Among children the inclusion of BCC was associated with a higher proportion of positive impacts for micronutrient intake, stunting, WLZ/WHZ, MUAC and anaemia. Programmes designed to improve these outcomes should aim to include BCC designed to address these diet and nutrition‐related issues to increase programme effectiveness. However, for other outcomes such as children's dietary diversity, LAZ/HAZ and wasting, the evidence on the effectiveness of integrating BCC in SAPs was less clear, although this could be a result of the considerable heterogeneity in BCC design and fidelity of implementation. It should be emphasized though that we did not attempt to categorize BCC activities according to duration, frequency, content, or quality; neither the frequency, content, nor quality of the BCC included was assessed and likely varied widely across programmes. A closer examination of the relationships between BCC activities and improvements in women's and children's nutrition outcomes is warranted.

The type and content of transfers needed for achieving nutrition outcomes is often debated and the relative effectiveness of different types of transfers is often context specific and can be outcome specific as well (Alderman et al., 2018). For example, in areas where markets are accessible and well‐stocked cash transfers or vouchers may be as effective or more so than in‐kind transfers for achieving nutrition and other social goals. Whereas, in areas with poor access to well‐stocked and regular markets, in‐kind transfers may be preferred, although this aspect of programme design is not a focus of this review. In our analysis, study arms that included both household and individual transfers were more likely to significantly increase Hb and reduce anaemia and stunting than those that did not. Likewise, study arms that included fortified foods were more likely to result in significant improvements in stunting, LAZ/WHZ, MUAC and anaemia.

In regard to wasting, the heterogeneity in programme design should be noted. Some of the studies included in this review address the role of transfers in preventing wasting, whereas others such as Grellety et al. (2017) and Bliss et al. (2018) confirm that cash transfers assist recovery from wasting when provided in the context of curative programmes. This curative approach to wasting differs from approaches to stunting, which being a cumulative indicator is likely to respond slowly once it occurs, if at all, and then more from changes in underlying determinants than specific targeted catch‐up interventions (Alderman & Headey, 2018). One study that compared various combinations of cash and supplements and which found that combining supplementary food and cash had a larger impact on weight for length or MUAC than cash or supplementary food alone focused only prevention during the 6‐month hungry season in Niger (Langendorf et al., 2014).

A few key findings have emerged from this study which we will highlight here. The first is that generally, SAPs can be used to improve diets among women and children. The second key finding revolves around the alignment of programme/transfer type to the diet or nutrition outcomes of interest. For example, for women's BMI and children's WLZ/WHZ, in‐kind programmes were generally more likely to lead to significant increases in these indicators as compared with cash transfer programmes. In regard to anaemia, of the programmes included in our study, none of the cash transfer or voucher programmes included were effective in improving Hb or reducing anaemia. Thus, to improve Hb or anaemia through SAPs, programme implementers and policymakers should consider using in‐kind transfers fortified with micronutrients or adding more anaemia specific design features to cash transfer and voucher programmes such as provision of micronutrient supplements or fortified foods (Tam et al., 2020), prevention and treatment of malaria (White, 2018), and treatment of helminth infections (Taylor‐Robinson et al., 2015) in places where these are prevalent. The third key finding from this review is that if programmes aim to reduce stunting and anaemia or to improve WLZ/WHZ, MUAC or Hb, they should strongly consider targeting women or children, including both household and individual transfers, fortified foods or products and BCC. The fourth key finding is that not all increases are necessarily positive and trade‐offs between improvements in some indicators and potential unintended negative effects need to be considered as was the case in the evaluation of the PM2A programme in Guatemala, which reduced stunting, but also increased women's BMI in a context with a high prevalence of stunting among children, and overweight and obesity among women.

The fifth key finding relates to the heterogeneity of the programme and evaluation designs. To clearly understand which programme design features are most important for achieving impacts, consistency in programme and evaluation design across several programmes in different contexts is needed. The example illustrated for programme impact on wasting in which some programmes are designed to be preventative and others curative is one key example. Furthermore, to draw clear conclusions related to the inclusion of different design features, evaluation designs that randomize assignment of these features and compare the inclusion of the design feature(s) to a valid counterfactual are needed. Although this review revealed some possible trends in the relative effectiveness of programmes including or not including different design features, differences in effectiveness due to programme quality, contextual factors or potential to benefit or other factors cannot be ruled out, as the design features studied (targeting women and/or children, inclusion of BCC, household and individual rations, and fortified food/supplement) were not randomly assigned in the vast majority of included studies. Very few studies have done this. One recent meta‐analyses included seven studies conducted between 2000 and 2021, which assessed the impact of BCC in conjunction with transfers compared to transfers alone and claim that BCC does not significantly improve anthropometric measures relative to cash alone (Little et al., 2021). However, there are methodological nuances that were not considered in that review. For example, the study from Myanmar by Field and Maffioli (2021) included in the meta‐analysis rejects the equality of the cash plus BCC and the cash only treatment effects on stunting (*p* = 0.02) in a regression that include covariates, whereas the study by Little et al. (2021) converts the means into an odds ratio and finds no significant difference at *p* < 0.05. Similarly, although Ahmed et al. (2019) find a difference in height for age between cash transfers with and without BCC in Bangladesh that is significant at the 1% level in regression analysis, Little et al. (2021) do not report a significant difference for the means from this study in the forest plots they illustrate. Thus, even when randomized studies are available to assess the effectiveness of including different design features, conclusions may vary based on the approaches used in summarizing/analysing the data.

Lastly, there is still a paucity of evidence related to the cost‐effectiveness of SAPs, effectiveness of SAPs for improving micronutrient status and preventing an increase in the prevalence of overweight and obesity for all populations and for improving diet and nutrition outcomes among other populations such as men, children 5–19 years of age and the elderly. A cost study of two of the programmes included in this review revealed that the programme variations that were the most intensive (PM2A programme in Guatemala that provided the full family ration + CSB and the PM2A programme in Burundi that provided benefits for the full 1000 days) were the most cost‐effective for reducing stunting. Additionally, the evaluation of these programme revealed that some of the programme variations, although less expensive, were not effective for reducing stunting which was the primary objective of those programmes (Heckert et al., 2019). Further research in these areas is urgently needed as many countries are increasing their investments in SAPs, and if intentionally designed, these programmes can do much more than reduce poverty and food insecurity. They can be used to improve diet and nutrition outcomes which could lead to extensive returns on these investments.

## AUTHOR CONTRIBUTIONS

The scope of this study was developed by Deanna K. Olney, Aulo Gelli, Neha Kumar, Harold Alderman and Ara Go, and built on a review initiated by Ahmed Raza and colleagues, and completed in collaboration with Deanna K. Olney, Aulo Gelli, Neha Kumar, Harold Alderman and Ara Go. The review was conducted by Deanna K. Olney, Aulo Gelli, Neha Kumar, Harold Alderman and Ara Go. The paper was drafted by Deanna K. Olney, Aulo Gelli, Neha Kumar, Harold Alderman and Ara Go. Ahmed Raza provided feedback throughout the initial review process and on drafts of the paper. All authors have read the final version of the paper and agreed to its content.

## CONFLICT OF INTEREST

The authors declare no conflict of interest.

## Supporting information

Supporting information.

## Data Availability

All data used for this paper is available in the supplemental tables. No new data sets were created.
