# Supplementary material for: Social assistance programme impacts on women's and children's diets and nutritional status
Source: Matern Child Nutr. 2022 Jun 20;18(4):e13378. doi: 10.1111/mcn.13378 (PMC9480902; doi:10.1111/mcn.13378)
Supplement: Supplementary file 1 — Supporting information. [file MCN-18-e13378-s001.docx]

**Appendices**

**Supplemental Table 1: Search topics and terms used**

| **Topic** | **Terms** |
| --- | --- |
| **Program type** |  |
| Social protection (general) | “social protection” OR “safety net*” OR “social assist*” |
| Cash transfers | “cash transfer” |
| In-kind transfers | “in-kind transfer*” OR “food transfer*” OR (“food aid*” AND program) OR (“food assist*” AND program) |
| Vouchers | voucher |
| **Diet** | diet* OR “infant feeding” OR “child feeding” OR micronutrient* OR “food consumption” OR calorie |
| **Nutrition** | anthropom* OR BMI OR “body mass index” OR stunting OR wasting OR underweight OR overweight OR obesity OR anemia |

**Supplemental Table 2: Search results^†^**

| **Program type** | **Articles identified** | **Remaining after title and abstract review (and after consultations)** | **Publications included** | **Unique program evaluations included^1^** | **Study arms included** |
| --- | --- | --- | --- | --- | --- |
| Cash transfers | 119 | 66 | 21^‡^ | 18 | 23 |
| In-kind transfers | 24 | 13 | 16^§^ | 10 | 21 |
| Vouchers | 63 | 11 | 4 | 4 | 4 |
| Social protection / Safety nets | 114 | 31 | Relevant studies added to other categories as appropriate | N/A | N/A |
| Social assistance | 15 | 0 | Relevant studies added to other categories as appropriate | N/A | N/A |
| Food aid | 48 | 17 | Relevant studies added to other categories as appropriate | N/A | N/A |
| Food assistance | 175 | 28 | Relevant studies added to other categories as appropriate | N/A | N/A |
| **Total** | **558** | **166** | **36**^‡^ | **32** | **48** |

^†^Several evaluations had multiple treatment arms as such they could fall under more than one category depending on the treatment arms (for example, the same evaluation could be in both the cash and in-kind categories; ^‡^includes studies identified with other search terms such as social protection or safety nets; ^§^includes studies identified with other search terms such as food aid or food assistance

**Supplemental Table 3: Proportion of program evaluations and study arms, total and by program type, by region^†^**

|  | **Program Evaluation (%)** | | | |  | **Study Arm (%)** | | | |
| --- | --- | --- | --- | --- | --- | --- | --- | --- | --- |
|  | **Total** | **Cash** | **In-kind** | **Voucher** |  | **Total** | **Cash** | **In-kind** | **Voucher** |
|  | **n=32** | **n=18** | **n=10** | **n=4** |  | **n=48** | **n=23** | **n=21** | **n=4** |
| **Africa** | 46 | 33 | 60 | 25 |  | 42 | 30 | 57 | 25 |
| **Latin America and the Caribbean** | 32 | 39 | 30 | 25 |  | 40 | 43 | 38 | 25 |
| **South Asia** | 7 | 11 | 10 | 25 |  | 10 | 13 | 5 | 25 |
| **East Asia and the Pacific** | 11 | 17 |  |  |  | 6 | 13 |  |  |
| **Middle East and North Africa** | 4 |  |  | 25 |  | 2 |  |  | 25 |

^†^ Some evaluations fall in more than one category for example in both the cash and in-kind categories, thus the total number of evaluations is smaller than the combined total across cash, in-kind and vouchers. However, the number of study arms is unique by program type.

**Supplemental Table 4: Program components and impacts of cash transfer programs on women’s diet and nutrition outcomes by study group^†^**

|  | **Program design features** | | | | | |  | **Program impacts** | | | | | |
| --- | --- | --- | --- | --- | --- | --- | --- | --- | --- | --- | --- | --- | --- |
|  | **Condition** | **Target** | **Transfer** | | | **BCC** |  | **Diet** | | **Anthropometric** | | **Biochemical** | |
| **Country (reference)**  **Study arm** |  | **W or C** | **Cash** | **Food**  **(UF or FF)** | **HH + Individual** |  |  | **DD** | **MN intake** | **BMI** | **MUAC** | **Hb** | **Anemia** |
| **Burkina Faso** (Houngbe et al., 2019) |  | x | x |  |  |  |  | + |  |  |  |  |  |
| **Somalia** (Grijalva-Eternod et al., 2018) |  | x | x |  |  |  |  | + |  |  |  |  |  |
| **Nepal** (Saville et al., 2018) |  | x | x |  |  | x |  | + |  | + | n |  |  |
| **Pakistan** (Fenn et al., 2017) |  |  |  |  |  |  |  |  |  |  |  |  |  |
| Double cash |  |  | x |  |  | x |  |  |  | n | n | n | n |
| Standard cash |  |  | x |  |  | x |  |  |  | n | n | - | n |
| **Ecuador** (Schady, 2012) |  | x | x |  |  |  |  |  |  |  |  | + | + |
| **Mexico** (Cunha, 2014) | BCC | x | x |  |  | x |  |  | +^‡^ | n |  |  |  |
| **Study arms (n)** | **BCC=1** | **5** | **7** |  |  | **4** |  | **3** | **1** | **4** | **3** | **3** | **3** |
| **Positive impact (n)** |  |  |  |  |  |  |  | **3** | **1** | **0** | **0** | **1** | **1** |
| **No impact (n)** |  |  |  |  |  |  |  | **0** | **0** | **4** | **3** | **1** | **2** |
| **Negative impact (n)** |  |  |  |  |  |  |  | **0** | **0** | **0** | **0** | **1** | **0** |

^†^positive (+), neutral (n), negative (-); ^‡^positive impact on vitamin C intakes while neutral impact on iron and zinc intakes. Abbreviations: BCC, behavior change communication; BMI, body mass index; C, children; DD, dietary diversity; FF, fortified food; Hb, hemoglobin concentration; HH, household; MN, micronutrient; MUAC, mid-upper arm circumference; UF, unfortified food; W, women.

**Supplemental Table 5: Program components and impacts of cash transfer programs on children’s diet and nutrition outcomes by study group^†^**

|  | **Program design features** | | | | | | | | |  | | **Program impacts** | | | | | | | | | | | |
| --- | --- | --- | --- | --- | --- | --- | --- | --- | --- | --- | --- | --- | --- | --- | --- | --- | --- | --- | --- | --- | --- | --- | --- |
|  | **Condition** | **Target** | **Child age** | **Transfers** | | | **BCC** | | |  | | **Diet** | | **Anthropometric** | | | | | | | **Biochemical** | | |
| **Country (reference)**  **Study arm** | **BCC, E, H** | **W or C** | **Pregnancy only, 1000 d, < 6 y, < 18 y, N/A** | **Cash** | **Food (UF, FF**^‡^**)** | **HH+I** | |  |  | | **DD** | | **MN intake** | **BW** | **HAZ** | **Stunted** | **WHZ** | **Wasted** | **MUAC** | **Hb** | | **Anemia** |  |
| **Brazil** (Assis et al., 2015) |  |  |  |  |  |  | |  |  | |  | |  |  |  |  |  |  |  |  | |  |  |
| First 6-month exposure | H, BCC | x | < 6 y | x |  |  | |  |  | |  | |  |  | n |  |  |  |  |  | |  |  |
| Last 6-month exposure | H, BCC | x | < 6 y | x |  |  | |  |  | |  | |  |  | n |  |  |  |  |  | |  |  |
| 12-month exposure | H, BCC | x | < 6 y | x |  |  | |  |  | |  | |  |  | + |  |  |  |  |  | |  |  |
| **Brazil** (Ford et al., 2020) | H, E |  | < 18 y | x |  |  | |  |  | |  | |  |  | n | n |  |  |  |  | | n |  |
| **Burkina Faso** (Houngbe et al., 2017, 2019; Tonguet-Papucci et al., 2017) |  | x | 1000 d | x |  |  | |  |  | | + | | + |  | n | n | n | n | n |  | |  |  |
| **Colombia** (Lopez-Arana, Avendano, Forde, et al., 2016; Lopez-Arana, Avendano, Van Lenthe, et al., 2016) | H, E | x | < 18 y | x |  |  | |  |  | |  | | + |  | n | n | n | + |  |  | |  |  |
| **DRC** (Grellety et al., 2017)^1^ |  | x | < 6 y | x | FF |  | | x |  | |  | |  |  | n | n | + |  | + |  | |  |  |
| **Ethiopia** (Gebrehiwot & Castilla, 2019) |  |  | N/A | x | UF |  | |  |  | |  | |  |  | n | n |  |  |  |  | |  |  |
| **Indonesia** (Kusuma et al., 2017) | H, E | x | N/A | x |  |  | |  |  | |  | |  |  |  | n |  | + |  |  | |  |  |
| **Indonesia** (Cahyadi et al., 2020) | H, E | x | < 18 y | x |  |  | | x |  | |  | |  |  |  | + |  |  |  |  | |  |  |
| **Mali** (Adubra et al., 2019)^1^ |  |  |  |  |  |  | |  |  | |  | |  |  |  |  |  |  |  |  | |  |  |
| SNACK+Cash | H | x | 1000 d | x |  | x | | x |  | | n | |  |  | n | n |  |  |  |  | |  |  |
| SNACK+Cash+LNS | H | x | 1000 d | x | FF | x | | x |  | | n | |  |  | n | n |  |  |  |  | |  |  |
| **Mexico** (Cunha, 2014) | BCC | x | N/A | x |  |  | | x |  | |  | | + |  |  |  | n |  |  |  | | n |  |
| **Mexico** (Raḿrez-silva et al., 2013) |  | x | < 6 y | x | FF | x | | x |  | |  | | + |  |  |  |  |  |  |  | |  |  |
| **Nepal** (Saville et al., 2018) |  | x | Pregnancy | x |  |  | | x |  | | n | |  | n | n |  | n |  |  |  | |  |  |
| **Niger** (Bliss et al., 2018) | BCC | x | N/A | x |  |  | | x |  | | + | |  |  |  |  | + |  | + |  | |  |  |
| **Pakistan** (Fenn et al., 2017) |  |  |  |  |  |  | |  |  | |  | |  |  |  |  |  |  |  |  | |  |  |
| Standard cash |  |  | < 6 y | x | FF |  | | x |  | |  | |  |  | + | + | n | n | n | n | | n |  |
| Double cash |  |  | < 6 y | x | FF |  | | x |  | |  | |  |  | + | + | + | + | n | n | | n |  |
| **Peru** (Andersen et al., 2015) |  |  |  |  |  |  | |  |  | |  | |  |  |  |  |  |  |  |  | |  |  |
| >= 2 years exposure | H, E | x | < 18 y | x |  |  | |  |  | |  | |  |  | n | + |  |  |  |  | |  |  |
| <2 years exposure | H, E | x | < 18 y | x |  |  | |  |  | |  | |  |  | n | n |  |  |  |  | |  |  |
| **Philippines** (Kandpal et al., 2016) | H, E | x | <18 y | x |  |  | | x |  | |  | |  |  | n | n^§^ |  |  |  |  | |  |  |
| **Somalia** (Grijalva-Eternod et al., 2018) |  | x | N/A | x |  |  | |  |  | | + | |  |  | n | n | n |  | n |  | |  |  |
| **Study arms (n)** | **BCC=5, E=7, H=12** | **21** | **Pregnancy=1, 1000 d=3, < 6 y =7, < 18 y= 6, N/A=5** | **22** | **UF=1, FF=5** | **3** | | **10** |  | | **6** | | **4** | **1** | **17** | **15** | **9** | **5** | **6** | **2** | | **4** |  |
| **Positive impact (n)** |  |  |  |  |  |  | |  |  | | **3** | | **4** | **0** | **3** | **4** | **3** | **3** | **2** | **0** | | **0** |  |
| **No impact (n)** |  |  |  |  |  |  | |  |  | | **3** | | **0** | **1** | **14** | **11** | **6** | **2** | **4** | **2** | | **4** |  |

^†^positive (+), neutral (n), negative (-); ^‡^fortified food or supplement; ^§^positive impact on severe stunting, but not stunting; Abbreviations. BCC, behavior change communication; BMI, body mass index; BW, birth weight; C, children; CCT, conditional cash transfer; DD, dietary diversity; E, education; FF, fortified food; H, health; HAZ, height-for-age Z-score; Hb, hemoglobin concentration; HH, household; I, individual; LNS, lipid-based nutrient supplement; MN, micronutrient; MUAC, mid-upper arm circumference; SNACK, Santé Nutritionnelle à Assise Communautaire dans la région de Kayes; UF, unfortified food; W, women; WHZ, weight-for-height Z-score.

**Supplemental Table 6: Program components and impacts of in-kind transfer programs on women’s diet and nutrition outcomes by study group^†^**

|  | **Program design features** | | | | |  | **Program impacts** | | | | | | | |
| --- | --- | --- | --- | --- | --- | --- | --- | --- | --- | --- | --- | --- | --- | --- |
|  | **Condition** | **Target** | **Food** | **HH+I** | **BCC** |  | **Diet** | | **Anthropometric** | | | | **Biochemical** | |
| **Country (Reference)**  **Study arm** | **H** | **W or C** | **(FF, UF)** |  |  |  | **DD** | **MN Intake** | **BMI** | **Underweight** | **Overweight** | **MUAC** | **Hb** | **Anemia** |
| **Burundi** (Leroy et al., 2016, 2020) |  |  |  |  |  |  |  |  |  |  |  |  |  |  |
| T24 |  | x | FF | x | x |  | + |  |  |  |  |  | + | + |
| T18 |  | x | FF | x | x |  | + |  |  |  |  |  | + | + |
| TNFP |  | x | FF | x | x |  | + |  |  |  |  |  | n | + |
| **Guatemala** (Leroy et al., 2019) |  |  |  |  |  |  |  |  |  |  |  |  |  |  |
| FFR+CSB | H | x | FF, UF | x | x |  |  |  | +^‡^ |  |  |  |  |  |
| RFR+CSB | H | x | FF, UF | x | x |  |  |  | n |  |  |  |  |  |
| NFR+CSB | H | x | FF | x | x |  |  |  | n |  |  |  |  |  |
| FFR+LNS | H | x | FF, UF | x | x |  |  |  | +^‡^ |  |  |  |  |  |
| FFR+MNP | H | x | FF, UF | x | x |  |  |  | +^‡^ |  |  |  |  |  |
| **Tanzania** (Fahey et al., 2019) | H |  | UF |  |  |  | n |  | n |  |  |  |  |  |
| **Nepal** (Harris-Fry et al., 2018; Saville et al., 2018) |  | x | FF | x | x |  | +^§^ |  | n^§^ | n^§^ |  | + |  |  |
| **Mexico** (Cunha, 2014) |  |  |  |  |  |  |  |  |  |  |  |  |  |  |
| Food alone or food combined |  |  | FF, UF |  |  |  |  | + | n |  | - |  |  |  |
| Food+BCC | H |  | FF, UF |  | x |  |  |  |  |  |  |  |  |  |
| **Study arms (n)** | **H=6** | **9** | **11** | **9** | **10** |  | **5** | **1** | **8** | **1** | **1** | **1** | **3** | **3** |
| **Positive impact (n)** |  |  |  |  |  |  | **4** | **1** | **3** | **0** | **0** | **1** | **2** | **3** |
| **No impact (n)** |  |  |  |  |  |  | **1** | **1** | **5** | **1** | **0** | **0** | **1** | **0** |
| **Negative impact (n)** |  |  |  |  |  |  | **4** | **1** | **3** | **0** | **1** | **1** | **2** | **3** |

^†^positive (+), neutral (n), negative (-); ^‡^negative effects at 12 months postpartum (but not at 18 or 24 mo postpartum); ^§^pooled results for men and women combined. Abbreviations: BCC, behavior change communication; BMI, body mass index; CSB, corn soy blend; DD, dietary diversity; FF, fortified food; FFR, full family ration; H, health; Hb, hemoglobin concentration; LNS, lipid-based nutrient supplement; MN, micronutrient; MNP, micronutrient powder; MUAC, mid-upper arm circumference; NFR, no family ration; RFR, reduced family ration; T18, Tubaramure program from pregnancy through 18 months of age; T24, Tubaramure program from pregnancy through 24 months of age; TNFP, Tubaramure program from birth through 24 months of age; UF, unfortified food.

**Supplemental Table 7: Program components and impacts of in-kind transfer programs on children’s diet and nutrition outcomes by study group^†^**

|  | **Program components** | | | | | | |  | **Program impacts** | | | | | | | | | |
| --- | --- | --- | --- | --- | --- | --- | --- | --- | --- | --- | --- | --- | --- | --- | --- | --- | --- | --- |
|  | **Condition** | **Target** | | **Transfer** | | | **BCC** |  | **Diet** | | **Anthropometric** | | | | | | **Biochemical** | |
| **Country (reference)**  **Study arm** | **BCC, E, H** | **W or C** | **Child age**  **Pregnancy only, 1000 d, < 6 y, < 18 y, N/A** | **Cash** | **Food**  **FF, UF** | **HH+I** |  |  | **DD** | **MN intake** | **BW** | **HAZ** | **Stunted** | **WHZ** | **Wasted** | **MUAC** | **Hb** | **Anemia** |
| **Burundi** (Leroy et al., 2016, 2018, 2020, 2021) |  |  |  |  |  |  |  |  |  |  |  |  |  |  |  |  |  |  |
| Tubaramure (pregnancy-24mo) |  | x | 1000 d |  | FF | x | x |  | n | +^‡^ |  | n | + | + | n |  | + | n |
| Tubaramure (pregnancy-18 mo) |  | x | 1000 d |  | FF | x | x |  | + ^‡^ | +^‡^ |  | n | + | + | + |  | + | + |
| Tubaramure (0-24mo) |  | x | 1000 d |  | FF | x | x |  | + ^‡^ | +^‡^ |  | n | n | n | n |  | + | n |
| **Guatemala** (Olney et al., 2018) |  |  |  |  |  |  |  |  |  |  |  |  |  |  |  |  |  |  |
| FFR+CSB | BCC, H | x | 1000 d |  | FF | x | x |  |  |  |  | + | +^§^ |  |  |  |  |  |
| RFR+CSB | BCC, H | x | 1000 d |  | FF | x | x |  |  |  |  | n | +^§^ |  |  |  |  |  |
| NFR+CSB | BCC, H | x | 1000 d |  | FF | x | x |  |  |  |  | n | n |  |  |  |  |  |
| FFR+LNS | BCC, H | x | 1000 d |  | FF | x | x |  |  |  |  | n | n |  |  |  |  |  |
| FFR+MNP | BCC, H | x | 1000 d |  | FF | x | x |  |  |  |  | n | +^§^ |  |  |  |  |  |
| **Mali** (Tranchant et al., 2019) |  |  |  |  |  |  |  |  |  |  |  |  |  |  |  |  |  |  |
| Any aid | E |  | N/A |  | FF |  |  |  | n |  |  |  |  |  |  |  |  |  |
| GFD |  |  | N/A |  |  |  |  |  | n |  |  |  |  |  |  |  |  |  |
| 2 forms of aid | E |  | N/A |  | FF |  |  |  | n |  |  |  |  |  |  |  |  |  |
| **Mali** (Adubra et al., 2019) |  |  |  |  |  |  |  |  |  |  |  |  |  |  |  |  |  |  |
| SNACK+LNS | H | x | 1000 d |  | FF |  | x |  | n |  | n | n | n |  |  |  |  |  |
| SNACK+LNS+cash |  | x | 1000 d | x | FF | x | x |  | n |  | n | n | n |  |  |  |  |  |
| **Malawi** (Gelli et al., 2017) |  |  | < 6 y |  | FF |  |  |  | + |  |  | n | n | + | n |  |  |  |
| **Mexico** (Raḿrez-silva et al., 2013) |  |  |  |  |  |  |  |  |  |  |  |  |  |  |  |  |  |  |
| Cash+children consumed FF |  |  | < 6 y | x | FF | x | x |  |  | + |  |  |  |  |  |  |  |  |
| **Mexico** (Cunha, 2014; Ramírez-Luzuriagac et al., 2016) |  |  |  |  |  |  |  |  |  |  |  |  |  |  |  |  |  |  |
| Food only (or pooled) |  | x | N/A |  | FF |  |  |  | + | + |  | n |  |  |  |  | n | n |
| Food+BCC |  | x | N/A |  | FF |  | x |  | n | n |  |  |  |  |  |  |  |  |
| **Nepal** (Saville et al., 2018) |  |  |  |  |  |  |  |  |  |  |  |  |  |  |  |  |  |  |
| PLA+Food | H | x | Pregnancy |  | FF | x | x |  | n |  | + | n |  | n |  |  |  |  |
| **Niger** (Brück et al., 2019) |  |  |  |  |  |  |  |  |  |  |  |  |  |  |  |  |  |  |
| RUTF |  | x | N/A |  | FF |  |  |  |  |  |  | n |  |  |  | n |  |  |
| RUTF+Food for Assets |  | x | N/A |  | FF | x |  |  |  |  |  | + |  |  |  | + |  |  |
| **Study arms (n)** | **BCC=5, H=7, E=2** | **15** | **Pregnancy=1, 1000 d=11, < 6 y =2, N/A=7** | **2** | **19** | **12** | **13** |  | **12** | **6** | **3** | **15** | **11** | **5** | **4** | **2** | **4** | **4** |
| **Positive impact (n)** |  |  |  |  |  |  |  |  | **4** | **5** | **1** | **2** | **5** | **3** | **1** | **1** | **3** | **1** |
| **No impact (n)** |  |  |  |  |  |  |  |  | **8** | **1** | **2** | **13** | **6** | **2** | **3** | **1** | **1** | **3** |

^†^positive (+), neutral (n), negative (-); ^‡^impact with CSB; ^§^impact at least one time point (1, 6, 9, 12, 18 or 24 mo of age). Abbreviations: BCC, behavior change communication; BW, birthweight; C, children; CSB, corn soy blend; DD, dietary diversity; E, education; FF, fortified food; FFR, full family ration; GFD, general food distribution; H, health; HAZ, height-for-age Z-score; Hb, hemoglobin concentration; HH, household; I, individual; LNS, lipid-based nutrient supplement; MN, micronutrient; MNP, micronutrient powder; MUAC, mid-upper arm circumference; NFR, no family ration; PLA, participatory learning approach; RFR, reduced family ration; RUTF, ready-to-use therapeutic food; SNACK, Santé Nutritionnelle à Assise Communautaire dans la région de Kayes; UF, unfortified food; W, women; WHZ, weight-for-height Z-score.

**Supplemental Table 8: Program components and impacts of voucher programs on women’s diet and nutrition outcomes by study group****^†^**

|  | **Program design features** | | | | |  | **Program impacts** | | | | | |
| --- | --- | --- | --- | --- | --- | --- | --- | --- | --- | --- | --- | --- |
|  | **Condition** | **Target** | **Transfers** |  | **BCC** |  | **Diet outcomes** | | **Anthropometric** | | **Biochemical** | |
| **Country (reference)**  **Study arm** |  | **W or C** | **Vouchers** | **HH+I** |  |  | **DD** | **MN Intake** | **BMI** | **MUAC** | **Hb** | **Anemia** |
| **Kenya** (Girard et al., 2017)  BCC+group+vouchers^‡^ | H | x | x |  | x |  | n | + |  | n | n | + |
| **Pakistan** (Fenn et al., 2017)  FFV^§^ |  | x | x |  | x |  |  |  | + | n |  |  |
| **Study arms (n)** |  |  | **2** |  | **2** |  | **1** | **1** | **1** | **2** | **1** | **1** |
| **Positive impact (n)** |  |  |  |  |  |  | **0** | **1** | **1** | **0** | **0** | **1** |
| **No impact (n)** |  |  |  |  |  |  | **1** | **0** | **0** | **2** | **1** | **1** |

^†^positive (+), neutral (n), negative (-); ^‡^intervention includes clinic-based BCC, community-based maternal support groups, and vouchers for OSFP vines, control group received BCC; ^§^cash value ~ US$14. Abbreviations: BCC, behavior change communication; BMI, body mass index; C, children; DD, dietary diversity; FFV, fresh food voucher; H, health; Hb, hemoglobin concentration; HH, household; I, individual; MN, micronutrient; MUAC, mid-upper arm circumference; SASHA, Sweet potato Action for Security and Health in Africa; W, women.

**Supplemental Table 9: Program components and impacts of voucher programs on children’s nutrition outcomes by study group^†^**

|  | **Program design features** | | | | | | |  | **Program impacts** | | | | |  | |
| --- | --- | --- | --- | --- | --- | --- | --- | --- | --- | --- | --- | --- | --- | --- | --- |
|  | **Condition** | **Target** | | **Transfers** | | | **BCC** |  | **Anthropometric** | | | | | **Biochemical** | |
| **Country (reference)**  **Study arm** | **BCC** | **W or C** | **Child age**  **1000 d, < 6 y** | **Vouchers** | **Food** | **HH+I** |  |  | **HAZ** | **Stunted** | **WHZ** | **Wasted** | **MUAC** | **Hb** | **Anemia** |
| **Honduras** (Siega-Riz et al., 2014) |  |  |  |  |  |  |  |  |  |  |  |  |  |  |  |
| vouchers+BCC+LNS^‡^ |  | x | 1000 d | x | FF | x | x |  |  |  |  |  |  | n | n |
| **Iran** (Ghodsi et al., 2018) |  |  |  |  |  |  |  |  |  |  |  |  |  |  |  |
| vouchers^§^ | BCC | x | < 6 y | x |  |  | x |  | n |  | n |  |  |  |  |
| **Pakistan, WINS**^¶^ (Fenn et al., 2017) |  |  |  |  |  |  |  |  |  |  |  |  |  |  |  |
| FFV^††^ |  | x | < 6 y | x |  |  | x |  | + | + | + | n | n | - | n |
| **Study arms (n)** | **BCC=1** |  | **1000 d=1, < 6 y = 2** | **3** | **FF=1** |  | **3** |  | **2** | **1** | **2** | **1** | **1** | **2** | **2** |
| **Positive impact (n)** |  |  |  |  |  |  |  |  | **1** | **1** | **1** | **0** | **0** | **0** | **0** |
| **No impact (n)** |  |  |  |  |  |  |  |  | **1** | **0** | **2** | **1** | **1** | **1** | **2** |
| **Negative impact (n)** |  |  |  |  |  |  |  |  | **0** | **0** | **0** | **0** | **0** | **1** | **0** |

^†^positive (+), neutral (n), negative (-); ^‡^Study assesses additional benefit of LNS so not included in analysis; ^§^vouchers had to be used at defined stores to receive food valued US $18; ^¶^Basic WINS program includes outpatient treatment, MN supplementation, BCC; ^††^value ~ US $14. Abbreviations: BCC, behavior change communication; BMI, body mass index; C, children; DD, dietary diversity; FF, fortified food; FFV, fresh food voucher; HAZ, height-for-age Z-score; Hb, hemoglobin concentration; HH, household; I, individual; LNS, lipid-based nutrient supplement; MUAC, mid-upper arm circumference; W, women; WHZ, weight-for-height Z-score; WINS, Women and Children/Infants Improved Nutrition in Sindh.
